# Hyper Expression of Mucin 5ac Indicates Poor Cancer Prognoses: A Meta-Analysis

**DOI:** 10.1097/MD.0000000000002396

**Published:** 2016-01-08

**Authors:** Xin Wang, Fei Yan, Run Shi, Xing Huang, Shiming Lu, Lin Xu, Binhui Ren

**Affiliations:** From the Fourth Clinical College of Nanjing Medical University, Nanjing, China (XW, FY, RS, XH, SL); Department of Thoracic Surgery, Nanjing Medical University Affiliated Cancer Hospital (XW, RS, LX, BR); and Jiangsu Key Laboratory of Molecular and Translational Cancer Research, Nanjing Medical University Affiliated Cancer Hospital, Nanjing, PR China (XW, FY, RS, XH, LX, BR).

## Abstract

The aim of the study was to explore the association between mucin 5ac expression and cancer prognosis.

A systematically comprehensive search was performed through PubMed, the Web of Science, and the China National Knowledge Infrastructure (CNKI). The prognostic value of mucin 5ac expression in cancer patients was evaluated.

The overexpression of mucin 5ac was found to be significantly associated with a poor prognosis in cancer patients (pooled HR: 1.53, 95%CI: 1.158–2.028, *P* = 0.003). This association was also detected in a biliary subgroup (pooled HR: 1.83, 95%CI: 1.269–2.639, *P* = 0.001) and a gastrointestinal subgroup (pooled HR: 1.44, 95%CI: 1.069–1.949 *P* = 0.017). In the geography subgroup analysis, a statistical association was found in the Asian subgroup (pooled HR: 1.69, 95%CI: 1.200–2.384, *P* = 0.003). In the clinical characteristics analysis, a statistical association was found between the hyper expression of mucin 5ac and lymphatic metastasis.

We indicated that mucin 5ac is a promising prognostic predictor for cancer, especially for biliary and gastrointestinal cancer, and is more suitable for predicting cancer prognoses in Asians.

## INTRODUCTION

Mucins are heavily glycosylated proteins that are expressed by epithelial cells of various organs.^1^ Some mucins enhance cancer cell proliferation through interacting with erbB1 EGFR and β-catenin,^2,3^ and the aberrant expression of mucins is associated with cancer development and poor prognoses.^4,5^ Mucin 5ac is a secretory mucin that has been shown to be highly expressed in various cancers.^6^ Some previous clinical studies have shown that mucin 5ac may be a useful prognostic predictor, and the hyper-secretion of mucin 5ac appeared to increase the risk of metastasis, thus influencing patient survival.^7^ However, the results are still inconclusive.^8^ Thus, we conducted this meta-analysis.

## MATERIALS AND METHODS

### Methods

The procedures performed in this meta-analysis are in accordance with recent guidelines for the reporting of meta-analyses (the PRISMA guidelines). And no ethical approval was needed because our meta-analyses were based on data from previously published studies.

### Data Sources and Searches

A computerized literature search was conducted of PubMed, the Web of Science, and the China National Knowledge Infrastructure (CNKI) with the following strategy: ([mucin 5ac or MUC5AC] AND [carcinoma or tumor or cancer] AND [prognosis or survival or outcome]) from 2000 to March 2015 in order to identify all studies that explored the association between mucin 5ac levels and cancer prognoses. Meanwhile, the time period, sample size, population, types of clinical trials, or types of reports on the retrieved studies were not limited, and we only reviewed articles in the English and Chinese languages. Every retrieved study was inspected manually for the inclusion criteria. To explore additional studies, we also examined the references of the included articles and reviews. The last search was carried out in May 2015.

### Study Selection

**FIGURE 1:**
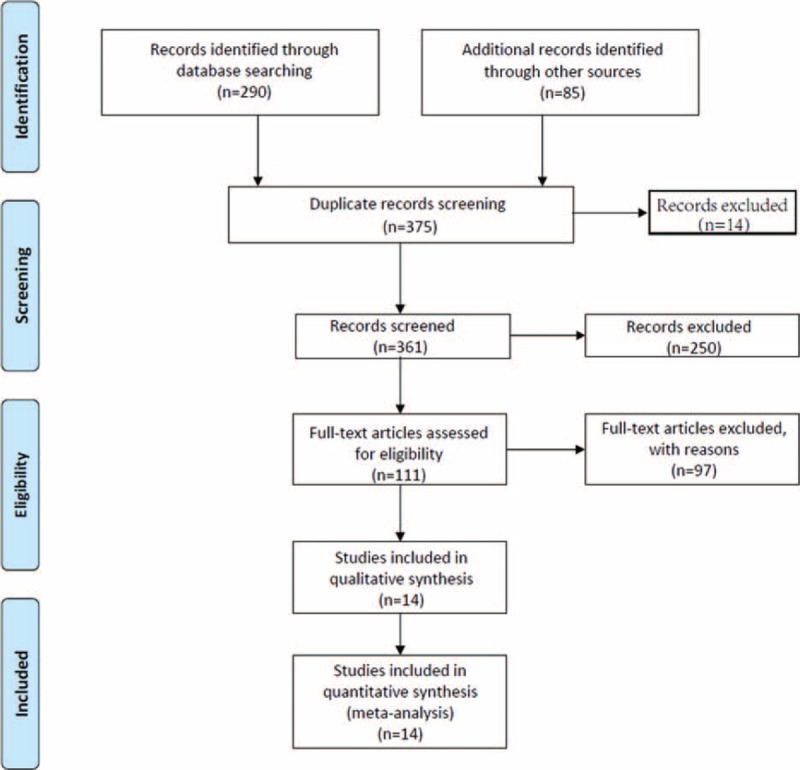
Flow diagram.

Publications were included in our analysis if they included a (1) proven diagnosis of cancer in humans, (2) an evaluation of the association between MUC5AC and cancer prognosis (patient survival data), (3) mucin 5ac quantity (protein or mRNA) analysis of the primary tumor (not in metastatic tumor or in tumor adjacent tissues) or in the serum, or an (4) eligible hazard ratio (HR) with 95% confidence intervals (CI) or other available data for estimating the HR with a 95%CI (including extracting data from K-M curves). We excluded non-RCTs, case series or case reports, review articles, editorials, letters, and comments. We also excluded non-English studies, nonhuman experiments, and studies with insufficient data. For overlapping studies, we took the largest study or the study with the first published samples. The study selection flow diagram is shown in Figure 1.

### Data Extraction and Quality Assessment

Two independent researchers (XW and XH) extracted data according to the inclusion criteria, and discordant studies were submitted to a third investigator (BR) for further review. To avoid overlapping patient populations, we compared data sources and geographic locations. The hazard ratios with the corresponding 95% confidence interval were extracted from every included study. For studies providing HRs, we obtained data directly.^8–16^ For studies that only provided K-M survival curves, we extracted the necessary data. In order to extract more robust data from the K-M curves, 2 methods were used ^17–21^ (using Engauge Digitizer 4.1, and by 2 independent researchers).^17–21^ We also collected the following data from each study: first author's name, year of publication, quantitative method, cancer type, sample size, country, cut-off value, specimen, and follow-up duration. Geography was categorized, based on the source country, as “Asian” or “non-Asian” (presented in Table 2). Each cancer type subgroup within 1 study was treated as a separate group in order to perform a cancer type-based subgroup analysis, and quantitative methods including IHC (immunohistochemistry), ELISA (enzyme-linked immunosorbent assay), and IB (Immunoblotting) were used to perform the subgroup analyses.

**TABLE 2:**
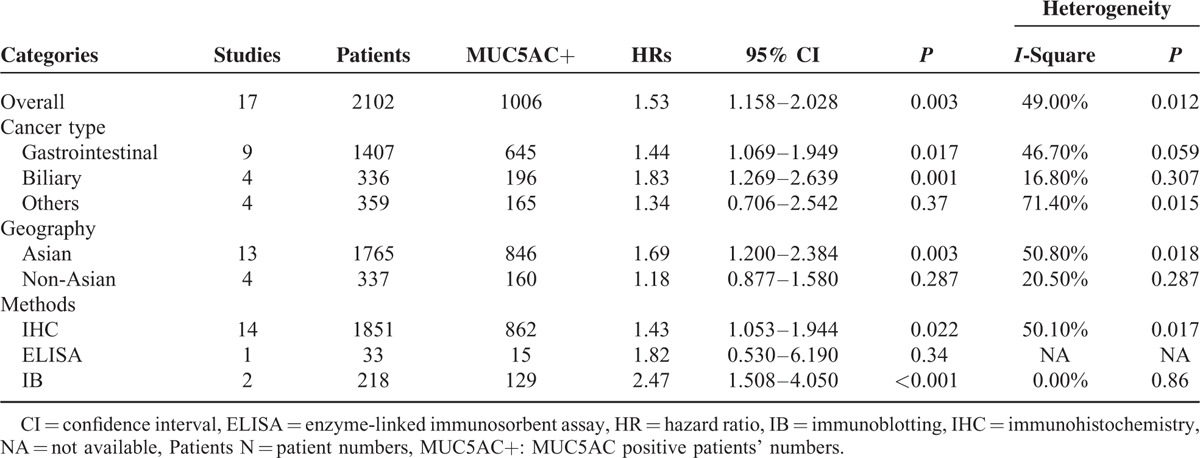
Main Results of Meta-Analysis

A quality assessment of the included studies was evaluated with the Newcastle–Ottawa quality assessment scale (NOS) ranging from 0 to 8 by 2 independent investigators (XW and FY). Studies with an NOS score ≥6 were considered high-quality studies. Studies from conference abstracts were defined as low-quality studies. Any inconsistencies were resolved by joint discussion.

### Statistical Analysis

HRs with 95% CIs were calculated to evaluate the relationships between mucin 5ac levels and overall survival. The heterogeneity among studies was assessed by calculating relevant *P* values and *I*^2^ values. If the *P* value was <0.05, indicating the presence of heterogeneity in studies, a random-effects model (based on the DerSimonian and Laird method) was used to assess the HRs and corresponding 95% CIs. Otherwise, the fixed-effects model (based on the Mantel–Haenszel method) was applied.^22–25^ An observed HR >1 indicated a worse prognosis in patients with mucin 5ac high expression and an HR <1 suggested a better prognosis. Thereafter, we performed subgroup analyses to explore sources of heterogeneity. A sensitivity analysis was performed by the sequential omission of individual studies. Publication bias was evaluated using a funnel plot and Egger's regression asymmetry test. Meanwhile, pooled odds ratios (ORs) with 95% CIs were calculated to describe the association between mucin 5ac expression and clinicopathological parameters. All statistical analyses were performed with STATA software (version 12.0, StataCorp, College Station, TX), and a *P*-value <0.05 was considered significant.

## RESULTS

### Description of Studies

The study selection processes using electronic database were presented in Figure 1. From an initial 361 potentially relevant articles, we excluded 14 duplicates and 250 irrelevant studies based on titles and abstracts. In the remaining 111 records, we reviewed the full texts and further excluded 97 studies for lacking of data. Finally, 14 articles (17 studies),^8–21,26^ 26 were included according to inclusion criteria. Imai and Shiratsu's studies included 2 different survival analyses separately (based on 2 different pathological types). Thus, a total of 17 studies, 2102 patients, were analyzed in this meta-analysis. All of the patients were pathologically diagnosed. Of all the eligible studies, 13 were conducted in Asian, whereas 4 in non-Asians areas, 9 were gastrointestinal cancers, 4 were biliary cancers, and 4 were other cancer types (including pancreatic cancer and nonsmall cell lung cancer). Details are shown in Table 1.

**TABLE 1:**
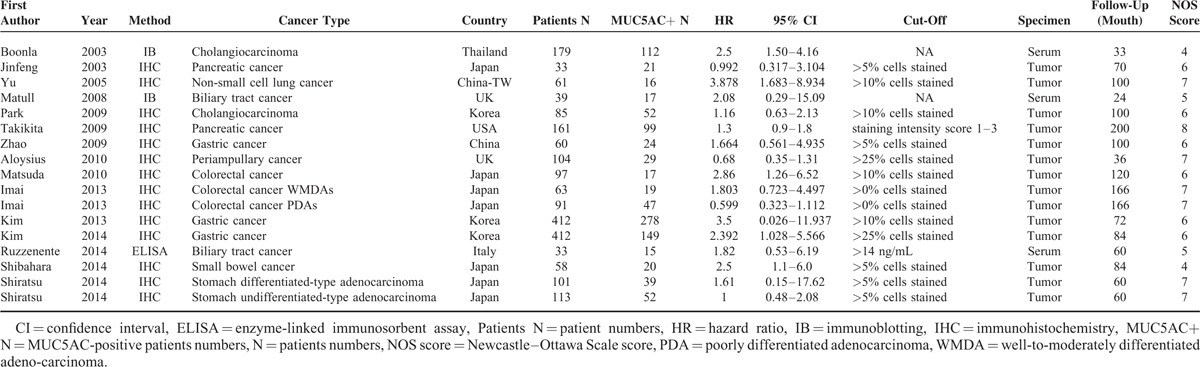
Main Characteristics of Studies Included in the Meta-Analysis

## OVERALL

**FIGURE 2:**
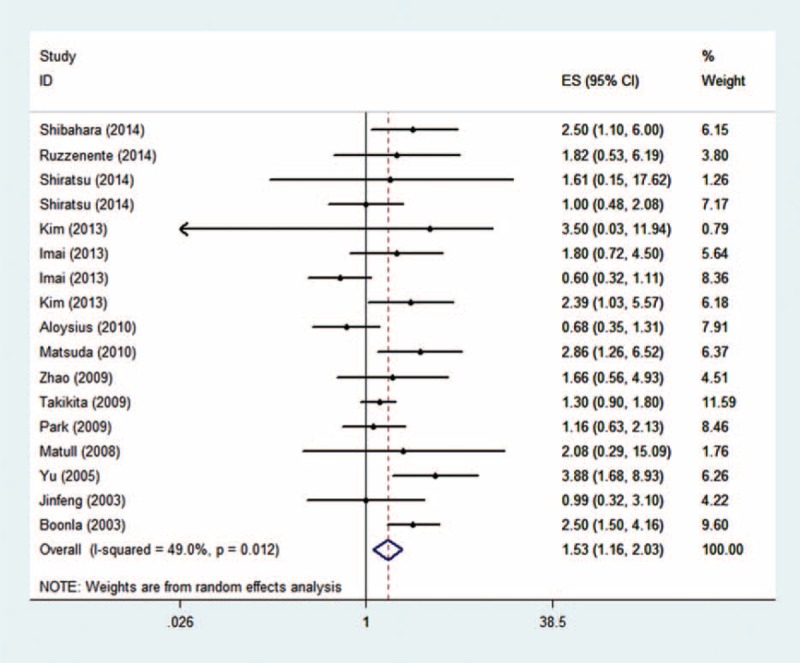
Overall meta-analysis.

A total of 2102 patients from 14 articles were enrolled in this analysis (pooled HR: 1.53, 95%CI: 1.158–2.028, *P* = 0.003, heterogeneity, *P* = 0.012, Figure 2). The results indicated that the overexpression of mucin 5ac was significantly associated with a poor prognosis in overall cancer.

### Subgroup Meta-Analysis

**FIGURE 3:**
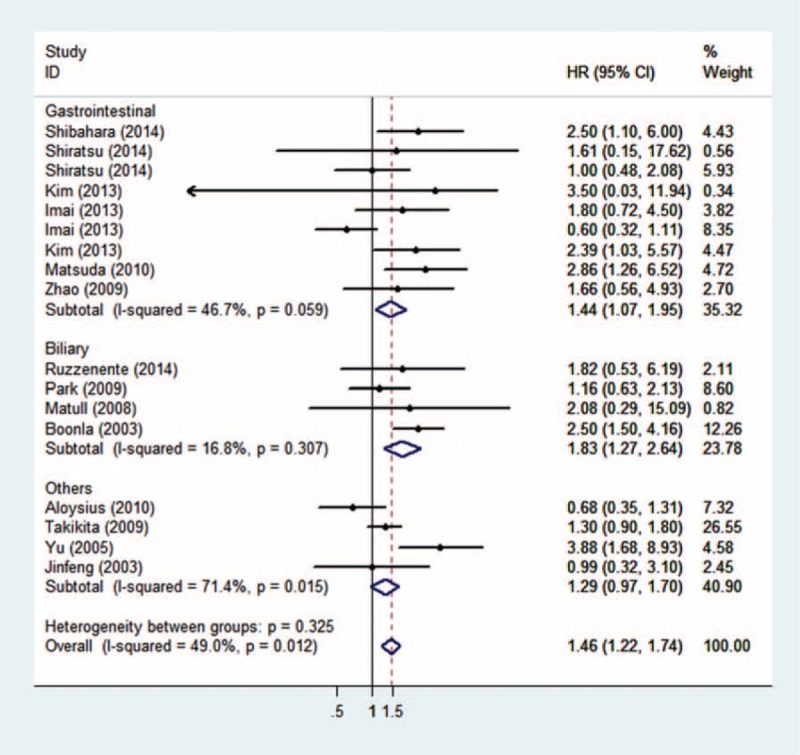
Meta-analysis based on cancer type.

**FIGURE 4:**
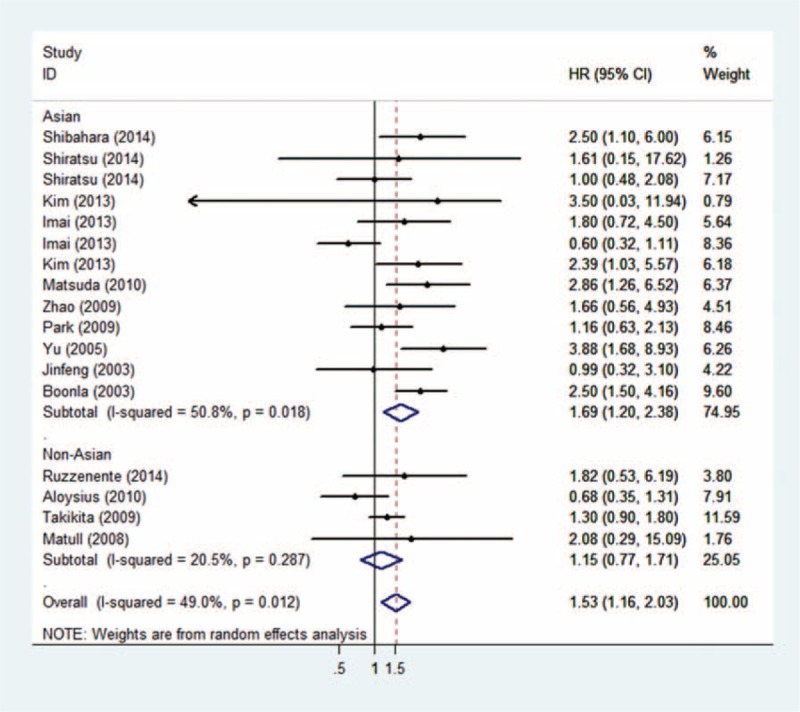
Meta-analysis based on geography.

As the *P* of heterogeneity was <0.05, heterogeneities within studies were considered. In order to explore heterogeneity, we performed the following subgroup analysis. For the cancer type subgroup analysis, patients with biliary carcinoma (pooled HR: 1.83, 95% CI: 1.269–2.639, *P* = 0.001) and gastrointestinal carcinoma (pooled HR: 1.44, 95%CI: 1.069–1.949, *P* = 0.017; details show in Figure 3) had poorer prognoses if they hyper-secreted mucin 5ac, and no heterogeneity was found within these groups (indicating that the cancer type difference might contribute to heterogeneity in the overall meta-analysis; details shown in Figure 3). Regarding geography, a significant association between high mucin 5ac levels and poor outcomes was found in Asian regions (pooled HR: 1.69, 95%CI: 1.200–2.384, *P* = 0.003 [details shown in Figure 4]), indicating that mucin 5ac may play a more important role in Asian populations. Finally, for the methods subgroup, a statistical association was observed in the IHC subgroup (pooled HR: 1.43, 95%CI: 1.053–1.944, *P* = 0.022), which was the predominant method used to detect mucin 5ac expression (see details in Table 2). As shown in supplemental Table 1, we found that the overexpression of mucin 5ac was significantly associated with lymphatic metastasis (pooled OR: 1.795, 95%CI: 1.147–2.810, *P* = 0.01).

### Publication Bias

**FIGURE 5:**
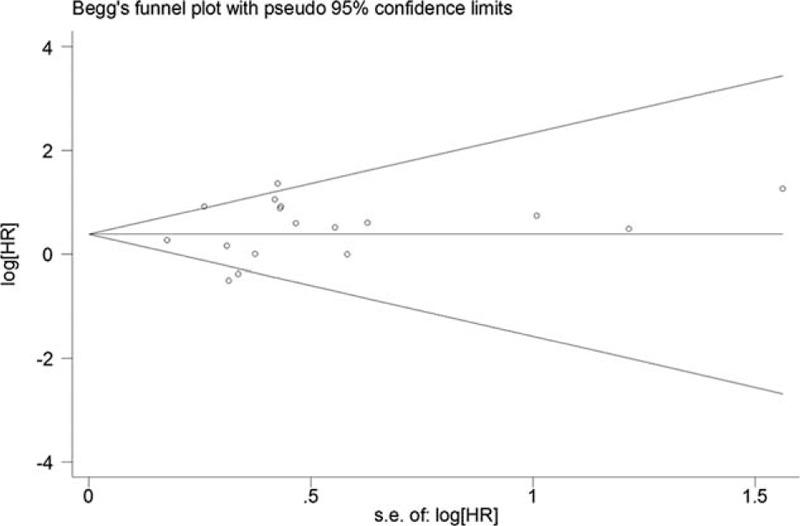
Begg's funnel plot.

Egger's test and Begg's funnel plot were used to assess publication bias among all studies. By Egger's test, *P* = 0.385 (the *P* values of Egger's tests were >0.05, indicating that no publication bias was found). No evidence of asymmetry was found in our funnel plot (Figure 5).

### Sensitivity Analysis

**FIGURE 6:**
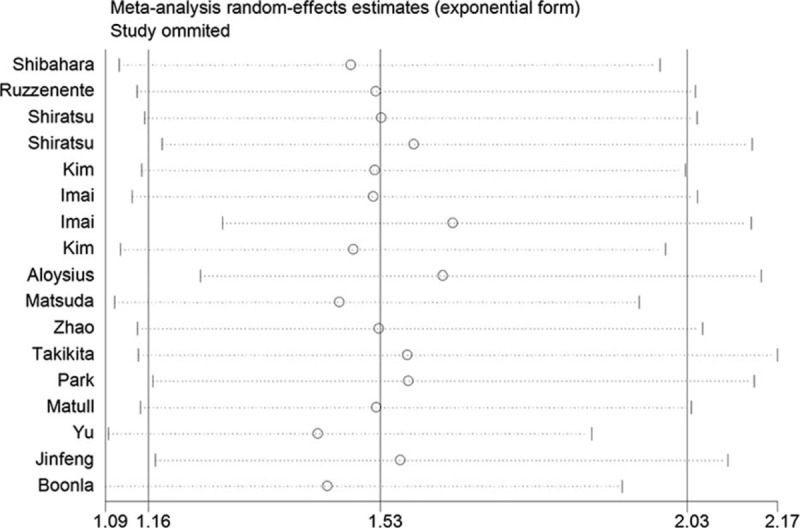
Sensitivity analysis.

To test the robustness of mucin 5ac expression and patient survival, a sensitivity analysis was performed by excluding the enrolled studies one by one and analyzing the effect and homogeneity of the remaining studies. The sensitivity analysis results showed no significant changes in the HRs when excluding any of the studies. The details are shown in Figure 6.

## DISCUSSION

### Summary of the Results

In the analysis of enrolled studies, we successfully drew some conclusions for clinical application. We observed that high mucin 5ac expression was significantly associated with a poor prognosis (pooled HR: 1.53, 95%CI: 1.158–2.028, *P* = 0.003). This indicates that mucin 5ac can be used for predicting cancer prognoses. In order to refine a more detailed conclusion, we stratified the analysis of enrolled studies. First, we performed a subgroup analysis by cancer type. Four studies were enrolled in the biliary carcinomas subgroup (pooled HR: 1.83, 95% CI: 1.269–2.639, *P* = 0.001) and 9 in the gastrointestinal carcinoma subgroup (pooled HR: 1.44, 95%CI: 1.069–1.949, *P* = 0.017). Both cancer types exhibited a significantly decreased survival rate in patients with hyper-expressed mucin 5ac. This may be because mucin 5ac can protect cancer cells from immune system attacks (the TRAIL-induced death pathways).^27^ However, researchers also noted that mucin 5ac has no effects on in vitro cell growth, cell survival, proliferation, or morphology,^28^ and studies on mucin 5ac function are relatively few. More function studies are needed to explore this finding. Second, in the Asian subgroup, 13 studies were included (pooled HR: 1.69, 95%CI: 1.200–2.384, *P* = 0.003) and demonstrated that mucin 5ac may play a greater role in Asian populations. In the non-Asian subgroup, 4 studies were included (pooled HR: 1.18, *P* = 0.287), which may be a result of a genotype difference and/or environmental exposure or small samples in the non-Asian subgroup. Third, to clarify the prognostic value of mucin 5ac expression detection methods, we found statistical significance in the IHC (pooled HR: 1.43, 95%CI: 1.053–1.944, *P* = 0.022) and IB (pooled HR: 2.47, 95%CI: 1.508–4.050, *P* < 0.001) subgroups, but we should also note that as the 3 studies that did not use IHC were all performed in biliary tract cancer patients, it is hard to draw conclusions about the IB subgroup. In the ELISA subgroup, we did not find a significant association, which may be because only 1 study was enrolled and included only 33 patients. Therefore, the major methods (IHC and IB) to detect mucin 5ac expression might be efficient for predicting cancer patients’ prognoses.

### Background

Cancer remains a major public health burden, accounting for 1 in 4 deaths in the United States.^29^ Identifying reliable and informative prognostic biomarkers for cancer patients in order to provide valuable information for clinical decision-making is of great interest.

Mucins are heavily glycosylated proteins that are expressed by various epithelial cell types existing in relatively harsh environments (the air–water interface of the respiratory system, the acidic environment of the stomach, the complex environment of the intestinal tract, and secretory epithelial surfaces of specialized organs such as the liver and pancreas^30,31^), which form a barrier that protects the epithelial cells.^31–33^ Tandem-repeats (TRs) are one of the hallmarks of mucins, which are rich in serine, threonine, and proline residues. Tandem-repeats are highly O-glycosylated and vital to mucin structure and function, and have been shown to be involved in specific ligand–receptor interactions.^1–3^ Mucins have also been shown to capture and hold biologically active molecules and antibodies, which, when released, might trigger inflammation, repair, or healing processes.^31,34^ Moreover, mucins are abnormally expressed in various cancers, and a previous report suggested that alterations in epithelial mucin core protein and glycosylation play an important role in cellular growth, differentiation, invasion, and immune surveillance for a variety of cancers,^31^ suggesting their potential role as promising biomarkers.^4,8,35,36^

Mucin 5ac, a secreted gel-forming mucin that is secreted by goblet cells, has been shown to be expressed in higher levels in adenocarcinomas than squamous carcinomas. The overexpression of mucin 5ac in cancer has been documented by other researchers ^37–39^ and has also been observed by us in one of our unpublished studies, with increasing occurrences of lymph node and distant metastasis and deeper invasion.^20,35,40^ Moreover, a 2013 study showed mucin 5ac could protect cancer cells from neutrophils’ attacking by suppressing TRAIL-mediated apoptosis .^27^ Therefore, mucin 5ac may serve as an important indicator in cancer prognosis.

## LIMITATIONS

Although this study is the first meta-analysis of the association between MUC5AC expression and patient survival, some limitations of this meta-analysis should be acknowledged. First, our meta-analysis did not include all human tumor types. Although mucin 5ac is a promising biomarker, the correlation still requires further research. For further confirmed results, large-scale studies are needed. Second, most of the included studies used IHC to determine mucin 5ac levels, and because it is difficult to follow the same protocol in every study, a technique bias may exist. Moreover, aberrant glycosylation of mucins is observed in various cancers, and this may influence antibody recognition,^41^ so more studies focused on the aberrant glycosylation of mucin 5ac are also needed. Third, we extracted data from survival curves in some enrolled studies because the survival data were not presented directly, and these calculated HRs and 95% CIs might be less reliable than the directly given data. Fourth, the applied methods for detecting mucin 5ac expression and the cut-off values were different in the enrolled studies, which could cause heterogeneity among the studies.

## CONCLUSIONS

This is the first meta-analysis evaluating the role of mucin 5ac as a cancer prognostic. Our study indicates that mucin 5ac may serve as a promising prognostic factor in cancer patients, especially in biliary carcinoma and gastrointestinal carcinoma patients, and is associated with lymphatic metastasis, which may play a more important role in Asian populations. More RCTs and functional studies are needed to explore the molecular mechanisms of mucin 5ac.
